# Tracking reproductive events: Hoof growth and steroid hormone concentrations in hair and hoof tissues in moose (*Alces alces*)

**DOI:** 10.1093/conphys/coad097

**Published:** 2023-12-13

**Authors:** Mandy J Keogh, Daniel P Thompson, John A Crouse

**Affiliations:** Division of Wildlife Conservation, Alaska Department of Fish and Game, 802 3rd St, Douglas, AK 99801, USA; Kenai Moose Research Center, Alaska Department of Fish and Game, 43961 Kalifornsky Beach Road Suite B, Soldotna, AK 99669, USA; Kenai Moose Research Center, Alaska Department of Fish and Game, 43961 Kalifornsky Beach Road Suite B, Soldotna, AK 99669, USA

**Keywords:** Alaska, Alces alces, cortisol, hair, hoof, keratin, moose, progesterone, stress

## Abstract

Measurements of reproductive and stress-related hormones in keratinous tissues (e.g. hair, claws, hooves, baleen) can provide a record of stress and reproductive response in wildlife. We evaluated a method to collect keratin tissue from hooves of immobilized moose (*Alces alces*) and validated enzyme immunoassays for measuring cortisol and progesterone in hooves and hair. We also measured the annual growth and wear rates of moose hooves. Progesterone (range: 1.0–43.7 pg/mg) and cortisol (range: 0.05–2.9 pg/mg) were measurable and showed variation among hoof samples and moose. Pregnant females had twice as high progesterone concentrations (18.00 ± 3.73 pg/mg) from hoof sample locations post breeding compared to non-pregnant moose (9.40 ± 0.25 pg/mg). Annual hoof growth differed between the front (5.58 ± 0.12 cm) and rear (4.73 ± 0.13 cm) hooves and varied by season with higher growth rates during summer which decreased into autumn and winter. Adult female hooves represented between 1.6 and 2.1 years of growth and included up to two reproductive cycles. We established a method to estimate hoof growth rate and applied this to postmortem samples and were able to detect previous pregnancies. Shoulder guard hairs grew between August and March including during late gestation; however, hair progesterone concentrations (range: 2–107.1 pg/mg) were not related to reproductive state. Hair cortisol concentrations in our study (range: 0.2–15.9 pg/mg) were within the range of values previously reported for cervids. Our study supports the use of hooves for longitudinal sampling and measuring reproductive and stress-related hormones, providing a new tool for tracking reproductive events and understanding what variables may contribute to population level changes in reproduction.

## Teaser

We describe the growth rate of hooves and methods to collect multiple hoof tissue samples from live and postmortem moose. We measured reproductive and stress-related hormones in hair and hoof samples and found progesterone concentrations in hooves varied with reproductive state, with progesterone concentrations in hooves identifying potential pregnancies.

## Introduction

Steroid hormone concentrations in metabolically active tissues (e.g. blood, feces) indicate reproductive state and stress in wildlife (Stewart *et al.*, 1985; Monfort *et al.*, 1993; Monfort *et al.*, 1995). However, these measures reflect recent responses that are affected by pursuit or handling of the animal (Omsjoe *et al.*, 2009; Thompson *et al.*, 2020) complicating the interpretation of these hormones. Fecal and urinary hormones can be used for determining pregnancy and eliminate the influence of acute stress providing a powerful method for non-invasive sampling (Creel *et al.*, 2009, Dulude-de Broin *et al.*, 2019b; Monfort *et al.*, 1993, Schwartz *et al.*, 1995), but sampling can be labor intensive and may require repeated sampling (Sheriff *et al.*, 2009; Pavitt *et al.*, 2016; Kamgang *et al.*, 2022; Høy-Petersen *et al.*, 2023). Wildlife biologists and managers are looking at innovative ways to improve their understanding of wildlife populations. Management and research operations have evolved over time with improved technology, efficiency and considerations for animal welfare. One area of research that has seen accelerated growth is wildlife endocrinology, with advancements in assays (e.g. Wasser *et al.* 2000) and the types of tissues that can be used for those assays (Sheriff *et al.*, 2011; Goheen and Jesmer, 2013).

The use of keratin tissue that incorporates reproductive and stress-related hormones as it grows over months or years has been beneficial for wildlife biologists assessing potential chronic stressors that may negatively influence the health, survival and reproduction of individuals and populations (Dulude-de Broin *et al.*, 2019b; Keogh *et al.*, 2022). These methods rely on incorporation of steroid hormones as the keratin tissue grows and hormone concentrations remaining metabolically unchanged once deposited. Hair has been the most common keratin tissue used to assess chronic stress and reproductive status in a variety of free-ranging wildlife (Bechshøft *et al.*, 2011; Macbeth *et al.*, 2012; Bryan *et al.*, 2013; Bryan *et al.*, 2014). The use of keratin tissue can also limit sampling and handling efforts on wild populations (Keogh *et al.*, 2021), utilize samples easily collected during harvests or other postmortem sampling (Hunt *et al.*, 2014; Spong *et al.*, 2020; Crain *et al.*, 2021; Davidson *et al.*, 2021; Keogh *et al.*, 2022) or as part of other ongoing sampling efforts of live animals (Dulude-de Broin *et al.*, 2019b; Roffler *et al.*, 2022) including cervids (Ventrella *et al.*, 2018; Madslien *et al.*, 2020; Ventrella *et al.*, 2020). Further, assessing both reproductive and stress-related hormones can provide a better understanding of what factors may be associated with reduced reproduction in wildlife (Bryan *et al.*, 2013; Dulude-de Broin *et al.*, 2019b; Keogh *et al.*, 2022). In mountain goats (*Oreamnos americanus*), higher predation risks had population level effects with greater fecal glucocorticoids and lower proportion of pregnancies (Dulude-de Broin *et al.*, 2019b). Reproduction in American marten (*Martes americana*) was positively influenced by diet (higher δ^15^N signature) and negatively related to fur cortisol concentration, highlighting the importance of protein in diet during late summer through fall while also capturing the separate relationship between reproduction and non-dietary stressors during the same period (Keogh *et al.*, 2022).

Moose are the largest member of the family Cervidae (true deer) along with elk (*Cervus canadensis*), red deer (*Cervus elaphus*), caribou (*Rangifer tarandus*) and black-tailed deer (*Odocoileus hemionus*), among others (Pitra *et al.*, 2004). Moose are an important subsistence resource, play a key role in shaping their habitat (McInnes *et al.*, 1992; De Vriendt *et al.*, 2021) and can be the focus of intensive management efforts and resources (Young Jr *et al.*, 2006; Boertje *et al.*, 2007). Hair cortisol concentrations in moose has been shown to relate to environmental and habitat variables, including temperature gradients and distance to wolf territories (Spong *et al.*, 2020). Hair cortisol concentrations were greater in moose with higher intensity of Deer Keds (*Lipoptena cervi*). The relationship between hair cortisol and body mass was non-linear: cortisol concentrations in hair increased with mass above 150 kg and decreased with mass below 125 kg (Madslien *et al.*, 2020). While these studies highlight the use of hair to assess the influence of potential stressors on moose, the molt and growth cycle of hair may prohibit accessing stressors and reproduction during late winter and early spring. In Alaskan moose, breeding occurs from mid-September to mid-October with gestation throughout winter and parturition occurring in May through June (Schwartz and Hundertmark, 1993; Laurian *et al.*, 2000). Moose hair is grown over several months with new hair growth beginning in May/June and continuing through November (Sokolov and Chernova, 1987), prior to gestation and winter, a potentially challenging period for moose. Given this mismatch in timing, we assessed the utility of hooves as an alternative to metabolically active tissues. If hormones are incorporated as hooves grow and concentrations change with physiological state, then hooves would support longitudinal sampling for reproductive and stress-related hormones. Keratin tissues, such as claws and whiskers, have demonstrated the potential for these tissues for assessing pregnancy and reproductive rates of individuals (Crain *et al.*, 2021; Keogh *et al.*, 2021), although non-linear growth or molting in some tissues (e.g. phocid whiskers) has complicated the use of these tissues in some species (Keogh *et al.*, 2020). These findings highlight the importance of understanding the variability in growth among seasons.

The objectives of our study were to (i) investigate the growth rate of moose hooves, (ii) validate enzyme immunoassays (EIA) to measure progesterone and cortisol in moose hoof and hair and (iii) to assess the utility of progesterone concentrations in moose hair and hoof samples to determine reproductive state in female moose.

## Materials and Methods

### Moose and sample collection

We studied captive moose at the Kenai Moose Research Center (MRC) and wild moose with known reproductive histories in the western lowlands (< 200 m elevation) of the Kenai Peninsula, Alaska, USA. The study area was comprised of mixed boreal forest interspersed with wetlands and lakes. Free-ranging captive moose (hereafter referred to as MRC moose, i.e. maintained on natural vegetation in 2.6 km^2^ enclosures) were held at the MRC operated by the Alaska Department of Fish and Game on the Kenai National Wildlife Refuge. All MRC moose had been non-reproductive for at least 1 year prior to the study. We immobilized female MRC moose (*n* = 7; Table 1) during late August 2018, December 2018, April 2019 and late August 2019 following the procedures outlined in Thompson *et al.* (2020). The female MRC moose were moved into a common pen with three bulls for breeding following the August 2018 sampling and were removed from the common pen at the end of October 2018. We collected blood samples in December 2018 and April 2019 to assess pregnancy status by analyzing serum for pregnancy specific protein B (PSPB, BioTracking, Moscow, ID, USA; Huang *et al.*, 2000, Sasser *et al.*, 1986). We marked and measured the hooves for growth and collected hoof samples and approximately 50 to 100 guard hairs with follicles from the dorsal portion of the shoulder above the scapular spine. Some samples also included undercoat but only the April 2019 samples had sufficient undercoat to measure hormones for all MRC moose. Additional hair samples were collected in December 2021 and March 2022. Hair and hoof samples were air dried and stored at room temperature until laboratory analysis. We weighed moose by walking animals across a platform scale (MP Series Load Bars; ± 0.2 kg; Tru-Test Limited, Auckland, NZ). All procedures for care, handling and experimentation were approved by the Alaska Department of Fish and Game Division of Wildlife Conservation Animal Care and Use Committee (protocols #0086 and #2014-17).

**Table 1:** Known age (years), body mass (kg), and pregnancy specific protein B (PSPB; ocular density values) in November/December 2018 and April 2019, and the final reproductive outcome from breeding efforts in fall 2018 for adult female moose (n = 7) at the Kenai Moose Research Center, Kenai Peninsula, Alaska, USA

ID	Age	Body mass (kg) Nov/Dec 2018	PSPB 12/5/2018	Body mass (kg) April 2019	PSPB 4/12/2019	Reproductive outcome
Cayenne	6/7	486	0.045	466	0.0865	Not pregnant
Minnie	10/11	442	0.6819	444	1.5988	Pregnant (twins)
Roxanne	9/10	498	0.9362	480	1.6574	Pregnant (twins)
Shiner	6/7	513	0.744	505	1.4245	Pregnant (singleton)
Sky	6/7	508	1.0002	492	1.5716	Pregnant (twins)
Stella	9/10	529	0.7119	509	1.3851	Pregnant (singleton)
Wilma	6/7	456	0.2802	427	0.0952	Not pregnant following an initial possible pregnant

Wild moose were collared and part of a long-term study providing reproductive information and cause of death (Table 2; D.P. Thompson, Alaska Department of Fish and Game, unpublished data). The reproductive histories were determined by a combination of PSPB values from blood collected in April and direct observation of a calf from an aircraft in spring (D.P. Thompson, Alaska Department of Fish and Game, unpublished data). Postmortem samples were collected from five wild female and one MRC moose. One front foot was removed at the knee or ankle joint and stored frozen at ≤ −20°C until further processing. Hair samples were collected from around the hoof using surgical scissors.

**Table 2:** Estimated age (years), reproductive histories, date and cause of death from wild (n = 5) and captive (n = 1) MRC moose on the Kenai Peninsula, Alaska, USA

ID	Age	Reproductive history	Date of death	Cause of death
B01	8	2016/17 Pregnant 2017/18 Pregnant 2018/19 Pregnant (not in hooves yet)	11/30/2018	Capture myopathy
B13	12	2017/18 Pregnant 2018/19 Pregnant	5/15/2019	Died at parturition
B33	4	2016/17 Pregnant 2017/18 Pregnant	5/26/2018	Died at parturition, retained placenta
B35	13	2017/18, no observation 2018/19 Unknown	5/3/2019	Brown bear
B38	18	2017/18 Pregnant 2018/19 Pregnant	5/6/2019	Apparent malnutrition, old animal
Flo	6	2016/17 Open 2017/18 Open	4/25/2018	Brown bear broke into enclosure

## Hoof growth and abrasion

**Figure 1:**
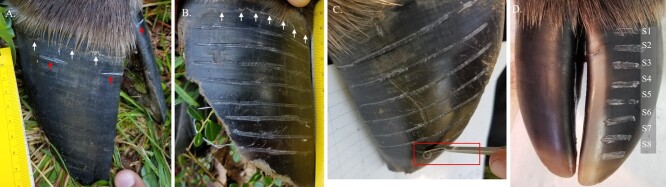
Photographs of a moose hoof during collection of hoof samples from live female moose at the Kenai Moose Research Center, Kenai Peninsula, Alaska, USA (A–C), and postmortem sampling in the lab (D). Notches (red lower arrows) on both the outer and inner digit on a front hoof to measure hoof growth (A) from the coronary band (white upper arrows). (B, C) Sampling hoof tissue for hormone analysis with V-shaped groover (C; box). Modified sample collection for the six moose sampled postmortem (D).

We marked both digits on one front and one rear hoof on the MRC moose by filing a V-shaped notch (2 cm long, 0.15 cm wide and 0.15 cm deep) into the hoof surface at the midpoint ~ 1 cm below the coronary band (Miller *et al.*, 1986; Sikarskie *et al.*, 1988; Tranter and Morris, 1992; Shelton *et al.*, 2012). We then measured the distance from the center point of the notch to the coronary band with calipers (Fig. 1A). Additionally, we measured hoof length of front and rear hooves by measuring perpendicularly from the coronary band to the end of the hoof edge along the medial edge of the digit, excluding digits with broken tips (*n* = 2). We remeasured in December 2018, April 2019 and August 2019 for hoof growth and hoof length by measuring the distance from the coronary band to the established V notch and hoof edge, respectively.

### Hoof Sample Collection

We cleaned the hoof surface with a clean, dry brush and by wiping it with 100% ethanol. We collected keratin samples from the outside digit of the front hoof. Using a V-shaped leather hand stitching groover (Weaver Leather Supply, Millersburg OH, USA), we removed a strip of hoof tissue (~6 cm long, 0.3 cm wide and 0.15 cm deep) parallel to the “growth band” (Fig. 1B, C). The mean distance from the coronary band to the midway point of each strip was measured with calipers (0.1 mm accuracy). In August 2018, we collected 8 hoof samples ~ 1 cm apart with S1 being 1 cm proximal to the coronary band (more recent growth) through S8 which was farthest from the coronary band (oldest tissue; Fig. 1B, C). Additional hoof and hair samples were collected following the methods above in December 2018 (S0), April 2019 (-S1) and August 2019 (-S2, -S3, -S4, -S5). We modified the sampling protocol for hoof samples collected postmortem based on the findings of the hoof growth and wear rates. Samples from postmortem hooves were collected from the medial portion of the hoof avoiding the side resulting in segments being about 2 cm in width and did not extend to the lateral part of the hoof (Fig. 1D).

## Progesterone and Cortisol

### Laboratory Validations

We validated commercially available enzyme immunoassay kits (EIA; Arbor Assays, Ann Arbor, MI, USA) for cortisol (K003) and progesterone (K025). Laboratory validations included recovery of added mass, parallelism and dilution linearity. Briefly, pools of methanol extracts from hair or hoof samples were serially diluted in assay buffer to determine the degree of parallelism to the standard curve. Results were plotted as the percentage bound vs the log of the relative dose, and slopes were visually inspected and compared for parallelism. The slope of the serially diluted pools from both tissues was linear and demonstrated parallelism to progesterone and cortisol standards (Supplementary Data S1). Assay accuracy was assessed by combining standards with an equal volume of pools, as determined for 50% binding in the parallelism assays. The diluted pools with and without standards added were then assayed and we plotted the results as the observed versus the expected concentrations and assessed the slope and y-intercept. The slopes for the observed versus expected dose for recovery of added mass (progesterone, cortisol) demonstrated a good fit for the y-intercept and the slope of the expected dose alone (Supplementary Data S1).

### Sample Preparation

Hoof samples were rinsed three times with 100% methanol, air dried and cut with surgical scissors and 20 mg (18.8 ± 2.8 mg) were weighed out in polypropylene tubes (Type I, Sarstedt®). For guard hairs, we removed visible contaminants with forceps, lined up individual guard hairs along the root end and removed hair follicles with surgical scissors. Guard hairs were then cut into 4-cm segments and placed into glass scintillation vials, washed three times with 100% methanol and air dried. Guard hair segments were further cut with surgical scissors and one to two samples of 20 mg (19.1 ± 2.4 mg) were weighted out in polypropylene tubes. Two 5-mm steel ball bearings (Retsch Inc, Newtown, Pennsylvania, USA) were added to each tube, and samples were pulverized at 30 KHz for 6 minutes (three times for hoof samples, two times for guard hair samples) using a Retsch MM 400 mixer mill with adapters for 10 vials (Verder Scientific Inc. Newtown, PA, USA).

### Hormone extractions and analysis

Hormones were extracted with 1.0 ml 100% methanol on a slow rotator for 24 hours at room temperature. Samples were then centrifuged at 15,493*g* for 13 minutes at 10°C and the supernatant was transferred to a new polypropylene tube. An additional 300 μl methanol was added to wash the pellets, then samples were centrifuged again and supernatant was removed and added to previous supernatant. Methanol supernatant (~1.3 ml) was stored at ≤ − 20°C.

Methanol extract was transferred to borosilicate glass tubes (300 μl for progesterone; 600–1000 μl for cortisol), dried under forced air and reconstituted in assay buffer specific for each kit. All samples were run in duplicate per manufacturer instructions and each assay included the full standard curve, non-specific binding, ‘zero’ blanks and two controls. The average intra-assay coefficient of variation for progesterone was 5.3% (range: 0–20.9%) in hoof samples and 5.8% (range: 0–19.6%) in hair samples, and the intra-assay coefficients of variation was 5.4% in hoof tissue and 5.7% in hair samples. For cortisol, the average intra-assay coefficient of variation was 3.6% (range: 0–11.7%) in hoof samples and 4.4% (range: 0–14.5%) in hair samples and inter-assay coefficients of variation was 11.3% in hoof tissue and 9.6% in hair samples.

## Statistical Analyses

We converted hoof growth and wear for each period (August–December; December–April; April–August) to daily rates by dividing each parameter by the days in each period for each individual animal. We estimated hoof wear by subtracting the hoof growth over one period from the difference between total hoof length at the end and beginning of that same period. We analyzed hoof growth rate, wear rate and hoof length using mixed model regression with individual as a random effect using programs in STATA version 15.0 (StataCorp LP, College Station, TX, USA). Using mixed model regression, we also analyzed progesterone and cortisol concentrations in hoof samples against the categorical variable for sample location (Fig. 4) for pre-breeding samples (S1, S2, S3, S4, S5, S6, S7, S8), and then also included the categorical variable pregnancy status (pregnant, non-pregnant) and the interaction with sample location for samples collected after breeding (S0, -S1, -S2, -S3, -S4, -S5). To minimize the effects of heteroscedacity and non-normal distributions, we used a robust sandwich estimator for the variance–covariance matrix for all mixed models (Rabe-Hesketh and Skrondal, 2008). All means reported are ± SE.

To determine time points associated with keratin samples from postmortem front hooves of moose, we assumed that hoof growth rate would follow the same annual fluctuations as body mass which is associated with seasonal rhythms in metabolism, food intake, forage quality and quantity (Schwartz *et al.*, 1984; Regelin *et al.*, 1985; Schwartz *et al.*, 1995). From 2012 to 2022, we collected body mass (*n* = 410) of adult female moose by weighing animals on a platform scale (MP Series Load Bars; ± 0.2 kg; Tru-Test Limited, Auckland, NZ). To establish a seasonal curve for body mass, first we considered Julian days as a circular statistic by converting Julian day (JD) to degrees and then radians (JD_radians). We then converted JD_radians to a sine curve (sine(JD_radians)). We then used mixed model regression, with individual as a random effect, to regress body mass against sine(JD_radians), which produced a statistically significant relationship (Supplementary Data S2; Wald $ \chi $^2^ = 259.15, *P* < 0.001, Y = −44.17X + 431.93). We then predicted daily body mass (bm) values for the entire year, and then generated daily rate of change in body mass (bm_t + 1_ − bm_t_). We corrected daily rate of change in body mass (range: −0.760437 kg/d to +0.760437 kg/d) to a positive value by adding 0.760437 kg/d as hoof growth is always positive. We then multiplied the daily change in body mass by daily growth metrics of hooves (55.58 mm/365 days = 0.1522 mm/d) and corrected the equation by adding a constant (0.037) to match similar annual hoof growth rates from measurements. Therefore, to generate daily hoof growth rates in the context of body mass change, we use the following equation:

Daily hoof growth rate = corrected daily rate of change in body mass • 0.1522 + 0.037.

We then regressed daily hoof growth rate against cosine (JD_radians) to create an annual curve (Supplementary Data S2) with the equation:

Daily hoof growth = −0.116X + 0.1525 where X = cos(radian(JD)).

Annual hoof growth using the estimated curve was 5.57 cm, compared with 5.58 ± 0.12 cm from actual measurements (Supplementary Data S3).

Progesterone and cortisol data (hair) were analyzed with Systat 13 (Systat Software, Inc., Point Richmond, CA). Normality was assessed with probability plots, and the data were log-transformed. We used a repeated-measures general linear model to compare hormone concentrations between hair segments within each sample and between samples collected in August 2018 to samples from August 2019. We removed progesterone concentrations that were below the detection limit (Stella Dec 2021 M3), or the percent binding was outside the standard curve (Minnie March 2022 M3). We report mean (± SE) for hormone concentrations.

**Figure 2:**
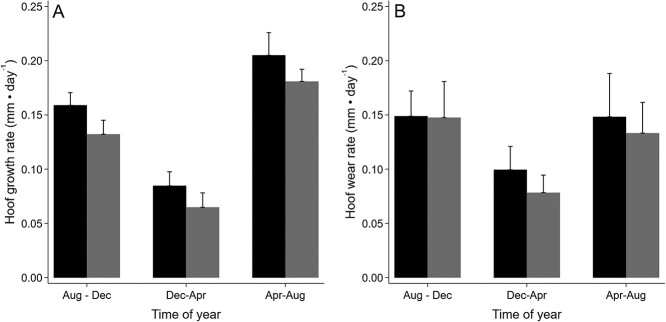
Hoof growth (A) and wear (B) rates (mm/day) during three time periods for the front and rear hooves of seven female moose at the Kenai Moose Research Center, Kenai Peninsula, Alaska, USA.

## RESULTS

### Pregnancy

PSPB values from December 2018 blood samples determined six of seven MRC moose were pregnant (ocular density values > 0.21; Table 1); however, one pregnant moose had a considerably lower PSPB value (0.2802; Wilma) and ultimately did not produce a calf. PSPB values in April 2019 confirmed that the two females with lower PSPB values in December were not pregnant (Table 1). The five pregnant MRC moose produced eight calves (two singleton, three twins) between May 3^rd^ and 27^th^. Reproductive histories of the wild moose, which correspond to the frame represented in the growth of the hooves at the time of death, are presented in Table 2.

### Hoof Length, Growth Rate and Wear Rate

Growth rate for the front and rear hooves varied by season (Wald χ^2^ > 103.05, *P* < 0.001), with higher growth rates during summer which decreased into autumn and winter (Fig. 2A; Supplementary Data S3). Similarly, hoof wear rates varied by season for the front and rear hooves (Wald χ^2^ > 50.62, *P* < 0.001), with lower wear rates during the winter (Fig. 2B). Annual hoof growth was different (Wald χ^2^ = 126.59, *P* < 0.001) between the front (5.58 ± 0.12 cm) and rear (4.73 ± 0.13 cm) hooves. Length of hooves were different during the three sample periods for both front (Wald χ^2^ = 21.67, *P* < 0.001) and rear (Wald χ^2^ = 15.02, *P* < 0.001) hooves; however, differences in total length were < 0.4 cm for front hooves and < 0.7 cm for rear hooves between sampling periods. Mean length of front hooves (9.59 ± 0.14 cm) was shorter than rear hooves (10.69 ± 0.15 cm; Wald χ^2^ = 85.09, *P* < 0.001).

## Progesterone and cortisol concentration in keratin tissues

### Hoof samples

For the MRC moose, no breeding occurred prior to autumn 2018 and all hoof segments collected in August 2018 (S1–S8) had a mean progesterone concentration of 9.1 ± 0.7 pg/mg (range: 2.9–27.3 pg/mg). Mean cortisol concentration during this period was 0.9 ± 0.07 pg/mg (range: 0.05–2.0 pg/mg). Progesterone and cortisol concentrations were not correlated (r = 0.161, *P* = 0.235) during the pre-breeding period. Progesterone and cortisol concentrations in post breeding samples (-S5, -S4, -S3, -S2, -S1 and S0) were not correlated in females that were pregnant (r = 0.100, *P* = 0.605) but there was a negative correlation between progesterone and cortisol concentrations for the two females that did not produce a calf (r = −0.609, *P* = 0.047).

**Figure 3:**
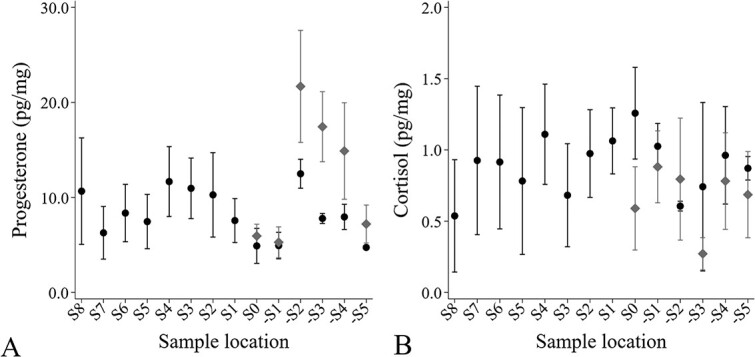
Progesterone (A) and cortisol (B) concentrations (pg/mg) from sample locations along the length of a moose hoof from seven adult female moose at the Kenai Moose Research Center, Kenai Peninsula, Alaska, USA. Segments were collected at 1 cm intervals from the proximal end of the hoof near the coronary band (S1) to the distal end (S8) in August 2018, prior the adult female moose being bred. One sample location was collected in December 2018 (S0) and April 2019 (-S1), while the remaining sample locations were collected in August 2019 (-S2, -S3, -S4, -S5). Predicted values with 95% confidence intervals from mixed-effect model regression.

Although progesterone concentrations in sample locations on moose hooves prior to breeding were significantly different (Wald χ^2^ = 13.00, *P* = 0.043), these differences were marginal (Fig. 3A). Progesterone concentrations in moose hooves after breeding were significantly different for the interaction between pregnancy status and sample location (Wald χ^2^ > 188.08, *P* < 0.001), with pregnant females having twice as high progesterone concentrations (18.00 ± 3.73 pg/mg) from hoof sample locations -S2, -S3 and -S4 compared to non-pregnant moose (9.40 ± 0.25 pg/mg; Fig. 3A). Cortisol concentrations in hoof segments prior to breeding were significantly different (Wald χ^2^ = 271.13, *P* < 0.001); however, these differences varied without any pattern with large confidence intervals (Fig. 3B). We did not find a significant interaction between sample location and pregnancy status for cortisol concentrations in moose hooves after breeding, but we did find that cortisol concentrations were significantly greater for non-pregnant moose (0.92 pg/mg) than pregnant moose (0.67 pg/mg; Wald χ^2^ = 9920.13, *P* < 0.001; Fig. 3B).

**Figure 4:**
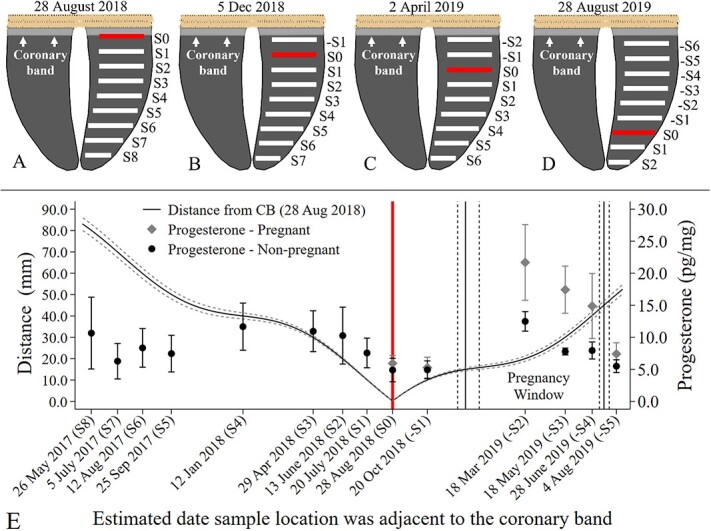
Illustration of sample locations (A–D) of samples collected (1 cm apart) from an individual moose hoof over one year period at the Kenai Moose Research Center, Alaska, USA. Hoof sample locations (S1–S8) collected on 28 August 2018, when sample location S0 was at the edge of the coronary band (A). In subsequent sampling periods (B, C, D) only new locations of hoof samples were collected if the sampling location was > 1 cm from the edge of the coronary band (e.g. 4B: sample location -S1 was < 1 cm from coronary band and not sampled). Estimated date that each hoof sample location (E) was adjacent to the coronary band with corresponding progesterone concentrations (Fig. 3A), based on a known fixed date (vertical line; sample location S0 at coronary band on 28 August 2018). Distance from coronary band (± 95% CI) for each sample location estimated from daily hoof growth (Supplemental Fig. 1B). Pregnancy window (vertical black line, ±95% CI vertical dashed line) determined by the dates when the hoof growth was 0.5 cm before (18 March 2019) and after (28 June 2019) when pregnant moose had elevated progesterone concentrations in hoof samples.

We applied the seasonal daily hoof growth rates calculated above for MRC moose (Supplementary Data S3) to determine time points associated with sample dates for the MRC moose to estimate when hoof samples collected would have been at the edge of the coronary band (Fig. 4A–D). S0 (collected in December 2018, Fig. 4B) would have been at the edge of the coronary band on 28 August 2018 (Fig. 4A). Pregnant MRC moose showed elevated progesterone values in hoof samples from time points between 8 March 2019 and 18 June 2019 (-S2, -S3, -S4). To determine a pregnancy window when moose should show elevated progesterone in the hooves associated with pregnancy, we determined the dates (± 95%CI) when the hoof growth was 0.5 cm before (8 March 2019) and after (18 June 2019) the elevated samples (Fig. 4E).

**Figure 5:**
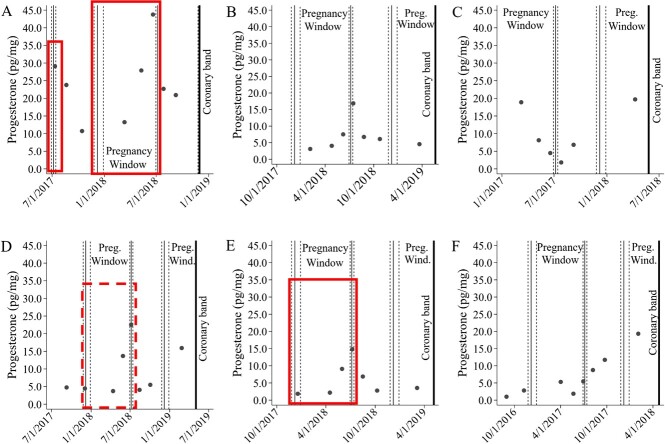
Progesterone concentrations (pg/mg) from sample locations along the length of a moose hoof and estimated date that each hoof sample location was adjacent to the coronary band from collected postmortem from five wild (A-E) and one captive (F) moose on the Kenai Peninsula, Alaska, USA. The pregnancy window is the estimated timeframe progesterone concentrations should be elevated if the moose was pregnant during that year. Pregnancy window (vertical black line, ±95% CI vertical dashed line) determined by the dates when the hoof growth was 0.5 cm before (18 March 2019) and after (28 June 2019). Boxes denote known pregnancies, and the dashed box (D) indicates a possible missed pregnancy.

Using the daily hoof growth rates calculated from MRC moose data above, we identified the pregnancy windows in the six postmortem moose hooves (Fig. 5). We captured elevated progesterone concentrations within the pregnancy windows in three moose (red boxes Fig. 5A, D, E) and possibly an earlier pregnancy in B01 (Fig. 5A). Four moose that died in April to May were pregnant but only had one hoof sample collected within the final pregnancy window (Fig. 5B–E) as most of the elevated progesterone would occur in the hoof tissues at the coronary band between March and June 2019. Moose B35 was not observed with a calf in the spring 2018 during parturition flights (Table 2); however, she had progesterone concentrations within the pregnancy window more than three times greater than progesterone concentrations outside the window suggesting she had been pregnant (Fig. 5D). One moose was not pregnant during the periods within her hoof (Flo, Fig. 5F, Table 2).

### Hair

Progesterone and cortisol were measurable in both guard hair and underfur samples. Mean progesterone concentration in guard hair segments was 23.1 ± 1.5 pg/mg (range: 2–107.1 pg/mg) and mean cortisol concentration was 3.1 ± 0.2 pg/mg (range: 0.3–15.9 pg/mg). Underfur was not present in all samples which prevented comparisons between sample dates. We measured progesterone and cortisol concentrations in underfur samples from MRC moose sampled in April 2019. Mean progesterone concentration in underfur was 9.8 ± 1.0 pg/mg (range: 5.3–12.6 pg/mg), while mean cortisol concentration was 1.5 ± 0.3 pg/mg (range: 0.7–2.9 pg/mg). For moose sampled postmortem, one hair sample was collected from around the hoof with mean progesterone concentration of 53.1 ± 38.5 pg/mg (range: 9.5–284.1 pg/mg) and mean cortisol concentration of 3.9 ± 1.6 pg/mg (range: 1.3–13.1 pg/mg).

Repeated measures comparing guard hair samples collected in August 2018 and 2019, both collected before breeding occurred, found there was a significant difference between years (*F*_1, 5_ = 6.786, *P* = 0.048) and segments (*F*_2,10_ = 16.690, *P* = 0.001) as well as a significant years and segments interaction (*F*_2,10_ = 10.336, *P* = 0.004) for progesterone. Post hoc analysis showed progesterone concentrations were greater in the Distal segment compared to Basal (*P* = 0.030) and M1 (*P* = 0.012). Cortisol concentrations did not differ between years (*F*_1, 5_ = 4.431, *P* = 0.089) or segments (*F*_2,10_ = 2.032, *P* = 0.182) nor was the interaction term, year*segment, significant (*F*_2,10_ = 0.276, *P* = 0.765).

**Figure 6:**
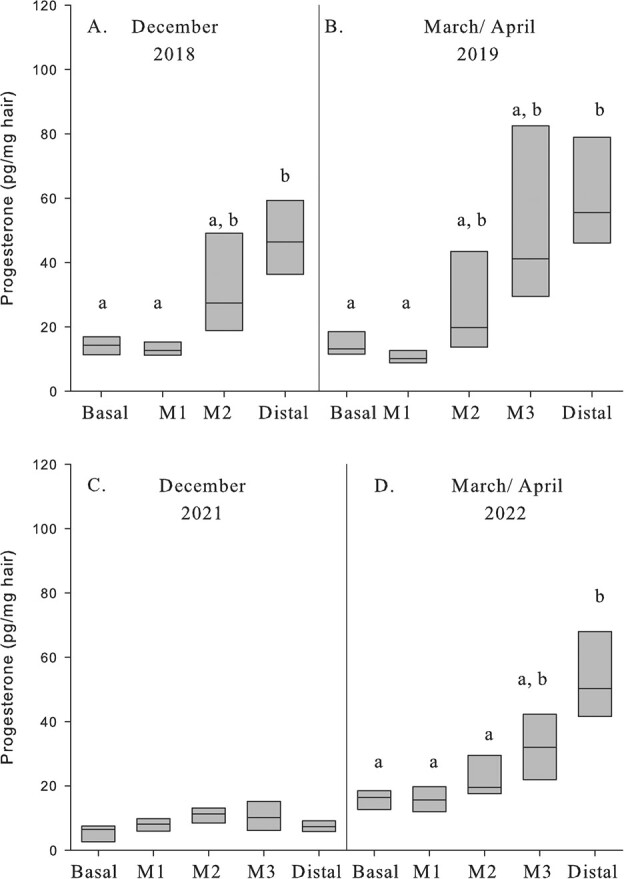
Progesterone concentrations (pg/mg) in guard hair segments from adult female moose sampled in December 2018 (A) and April 2019 (B) following breeding (5 pregnant, 2 non pregnant) and in December 2021 (C) and March/April 2022 when no breeding occurred at the Kenai Moose Research Center, Kenai Peninsula, Alaska, USA. Repeated measures found no difference between pregnant and nonpregnant moose in hormone concentrations. Different letters denote significant differences for progesterone concentrations between segments within each sample.

Progesterone concentrations in guard hair segments following breeding (December 2018) did not differ between reproductive state (*F*_1,5_ = 0.091, *P* = 0.775) but did vary among segments (*F*_3,15_ = 1.915, *P* = 0.002) and post hoc analysis found progesterone concentrations were greater in the distal segment compared to the basal and M1 segment (Fig 6A). Similar to December 2018, progesterone concentrations in April 2019 hair samples did not differ between reproductive state (*F*_1,5_ = 0.028, *P* = 0.875) while concentrations varied among segments (*F*_4, 20_ = 31.828, *P* < 0.001; Fig 6B).

We collected hair samples the following year when no breeding occurred (December 2021, March–April 2022). There was no difference in progesterone concentrations among segments in the December 2021 samples (*F*_3,15_ = 2.690, *P* = 0.084; Fig. 6C). Progesterone concentrations were significantly different among the segments in the March to April 2022 sample despite no breeding occurring (*F*_4,16_ = 18.099, *P* < 0.001; Fig. 6D). Post hoc analysis showed progesterone concentrations were greater in the distal segments in March to April 2022 (Fig. 6D).

**Figure 7:**
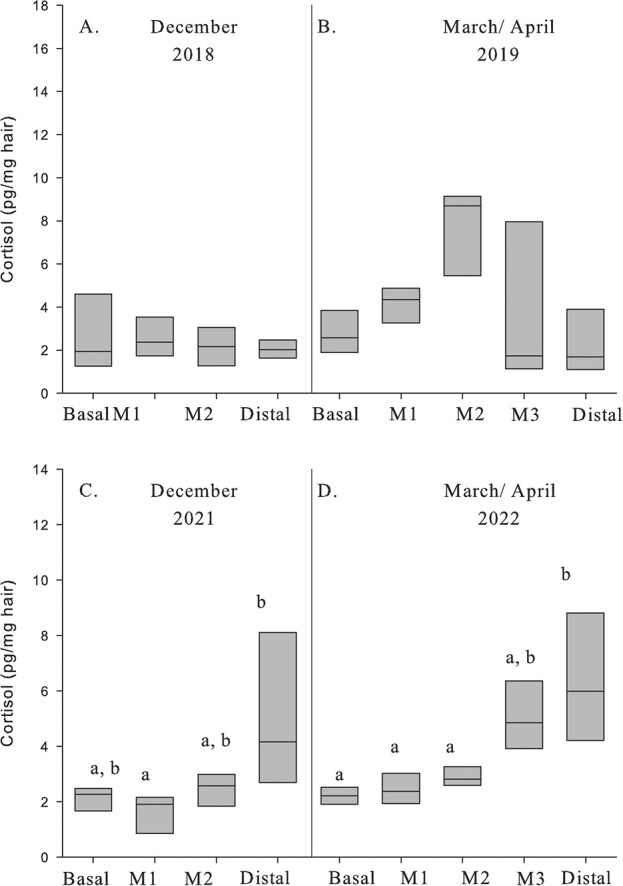
Cortisol concentrations (pg/mg) in guard hair segments from adult female moose sampled in December 2018 (A) and April 2019 (B) following breeding (5 pregnant, 2 non pregnant) and in December 2021 (C) and March/April 2022 when no breeding occurred at the Kenai Moose Research Center, Kenai Peninsula, Alaska, USA. Repeated measures found no difference between pregnant and nonpregnant moose in hormone concentrations. Different letters denote significant differences for cortisol concentrations between segments within each sample.

Guard hair cortisol concentrations did not differ between reproductive status following breeding (December 2018: *F*_1,5_ = 0.01, *P* = 0.914; April 2019: *F*_1,5_ = 0.265, *P* = 0.629; Fig. 7A, B) nor did concentrations differ among the segments (December 2018: *F*_3,15_ = 0.023, *P* = 0.995; April 2019: *F*_4,20_ = 1.841, *P* = 0.161; Fig. 7A, B). The following year, when no breeding occurred, there was a significant difference in cortisol concentrations among segments in December 2021 (*F*_3,18_ = 6.233, *P* = 0.004; Fig 7C) with the distal segment being greater than M1 (*P* = 0.028). There was also a significant difference among segments from the March to April 2022 samples (*F*_4,16_ = 18.099, *P* ≤ 0.001; Fig. 7D) with the distal segment being greater than basal, M1 and M2 segments (*P >* 0.031).

## DISCUSSION

We provide a method to collect hoof tissue using a V-shaped leather groover along the length of hooves from either live moose during chemical immobilization restraint or collecting samples postmortem. No damage or compromise to the hoof of the live moose was observed during or after our study. Of note is the ability of hooves to support repeated measurements of physiological parameters while limiting the sampling and handling of live wild moose. Serial samples of moose hooves could be collected postmortem from moose killed on roads or during liberal cow hunts as proposed for moose population with low nutritional status and predation pressure (Boertje *et al.*, 2007).

Understanding the growth rate of a tissue is necessary before physiological measures, such as steroid hormones or dietary stable isotopes, can be fully employed as a tool for monitoring wildlife populations. We measured the annual growth rate of moose hooves and developed a method to estimate when growth occurred based on the day of year hooves were sampled. Growth rates differed between the front and rear hooves, and each hoof represented between 1.6 and 2.1 years of keratin accumulation. Our findings are comparable to hoof growth rates of 6–8 cm per year (hoof keratin accumulation < 2 years) estimated for adult male moose based on stable isotope signatures (Kielland, 2001). However, our results are considerably lower than estimated hoof growth rate of 13.7 cm a year (hoof keratin accumulation 7–9 months) for moose from Isle Royal (Tischler *et al.*, 2019). We expect the growth estimates in our study to be applicable to female moose in Alaska. We documented that growth and wear rates of hooves varied seasonally and growth dramatically slowed over winter (December to April), a period during which resources are limited, the terrain is often covered with snow and during which most of gestation occurs (Schwartz and Hundertmark, 1993). The seasonal hoof growth rate estimates in our study are comparable to seasonal variation in growth rates in other cervids (Miller *et al.*, 1986) and will allow future studies to modify the sampling scheme.

### Hormones in hooves

We found pregnant moose had greater progesterone concentrations in hoof segments that were present at the coronary band between March and June, a period that corresponds to late gestation in moose (Laurian *et al.*, 2000; Boertje *et al.*, 2019). Fecal and blood progestogen concentrations in moose were elevated throughout gestation (~230 days) and associated with a rapid decline at parturition (Stewart *et al.*, 1985; Schwartz *et al.*, 1995). We captured the decline in progesterone at parturition within the pregnancy window for two MRC moose. The -S4 segment was estimated to be at the coronary band in June so that this tissue was grown near the end of gestation since all MRC moose in our study gave birth between May 3^rd^ and 27^th^. The decline in progesterone around parturition was also observed in moose sampled postmortem in our study.

In pregnant moose, progestogen concentrations increased between five and seven times in blood (Stewart *et al.*, 1985) and 4-fold in feces compared to non-pregnant moose (Schwartz *et al.*, 1995). However, we observed a 2-fold increase in progesterone concentrations in hoof tissue during gestation in moose, which is comparable to increases observed during pregnancy in Steller sea lion (*Eumetopias jubatus*) and northern fur seal (*Callorhinus ursinus*) whiskers, a continuously growing keratin tissue (Keogh *et al.*, 2021). While both otariid species had between a 2-fold and 3-fold increase in whisker progesterone concentrations during pregnancy, the whisker progesterone concentrations varied greatly between species (Steller sea lions: 6.1–145.5 pg/mg; northern fur seals: 1.4–477 pg/mg (Keogh *et al.*, 2021)). The growth rate of hooves in winter, when most of the gestation occurs, was much lower than during the rest of the year, and the reduced growth in hooves may have contributed to the lower hormone concentrations we observed in hooves compared to circulating progesterone concentrations during pregnancy. We propose progesterone concentrations within the pregnancy window can be used to classify reproductive state of female moose as follows: 18 pg/mg or greater indicates a pregnancy and 9 pg/mg or less indicates the female was not pregnant. Progesterone concentration between 10 and 17 pg/mg within the pregnancy window are considered marginal and comparison of progesterone concentrations within the pregnancy window to concentrations outside of the pregnancy window may allow for determination of reproductive state. For example, based on the 18 pg/mg pregnancy threshold, B38 was a false-negative. B38 did not have a progesterone concentration greater than 18 pg/mg within the pregnancy window, however, the progesterone concentration increased during the pregnancy window with progesterone concentrations more than doubling compared to the periods outside of the pregnancy window and a calf was observed during parturition surveys.

Several methods already exist for determining pregnancy or calf production in wild cervids. However, these methods often provide one time-point when samples (e.g. urine, feces) or observations (e.g. field cameras, parturition surveys) are collected and may not provide information on the drivers behind the reproductive outcomes. Further, there is often a narrow period during which these methods can be employed. For example, parturition flights are weather dependent and must occur during a limited period in the spring when calves are likely to be present. Our findings support the use of hoof tissue to assess pregnancy in moose, proving an additional tool for wildlife biologist and managers. Further, the sampling method outline in our study allows for repeated sampling of hooves, which are grown over multiple years incorporating one or two reproductive cycles depending on when the hooves were sampled.

Cortisol was measurable in hoof tissue, and we found variation in concentrations along the length of the hoof and among moose. Cortisol concentrations in hooves were greater in the non-pregnant MRC moose in our study and were negatively correlated with progesterone concentrations in non-pregnant moose only. Our study was small and interpretation of the relationship between cortisol and progesterone in the two females that failed to produce a calf after breeding requires caution. However, the methods described here and the use of hoof tissue for repeatedly measuring paired progesterone and cortisol concentrations may provide a better understanding of stress-induced suppression of reproduction in wildlife (Dulude-de Broin *et al.*, 2019b; Keogh *et al.*, 2022). For example, greater hair cortisol concentrations in cervids were found to be related to population densities (Franchini *et al.*, 2023), distance to wolf territories and temperature gradients (Spong *et al.*, 2020), parasite load and body mass (Madslien *et al.*, 2020). Future studies could assess if chronic stressors and the observed higher concentrations of cortisol in keratin tissues relate to the likelihood of pregnancy in wild populations of cervids.

### Hormones in hair

Progesterone and cortisol were measurable in underfur and guard hair collected from the shoulder region of adult female moose and in guard hairs around the hoof collected postmortem. Progesterone and cortisol were both about 3x greater in shoulder guard hair compared to underfur, similar to findings in Rocky Mountain goat hair samples (Dulude-de Broin *et al.*, 2019a).

We observed growth of shoulder guard hairs between August and March, including during gestation (December and March); however, progesterone concentrations in these hairs were not related to reproductive state in our study. We consistently found progesterone concentrations greater in the distal segments of guard hairs regardless of when sampled or the reproductive state of the moose. Similarly, progesterone concentrations were greatest in the distal portion of hair from Père David deer (*Elaphurus davidianus*) following their autumn molt (Ping *et al.*, 2017). Guard hair progesterone concentrations collected in our study in April 2019 from the shoulder area were slightly lower but comparable to hair samples from pregnant red deer collected in April from the neck (41.7–153.6 pg/mg hair) (Ventrella *et al.*, 2020).

The progesterone concentrations in hair around the hooves collected postmortem in our study had a larger range (9.5–284.1 pg/mg) compared to concentrations found in guard hair (2–107.1 pg/mg). The body area where the hair was collected may have contributed to the differences we observed (Macbeth *et al.*, 2010; Rakic *et al.*, 2023), potentially due to the variation in hair type and structure across the body and the molt progression in cervids (Cowan and Raddi, 1972; Sokolov and Chernova, 1987; Woods *et al.*, 2011; Rakic *et al.*, 2023). Further, previous studies have also reported outliers in progesterone and cortisol concentrations that were not able to be fully explained (Bryan *et al.*, 2013; Roffler *et al.*, 2022). The extreme progesterone concentration in our study (B1, 284.1 pg/mg) also had a high cortisol concentration (13.1 pg/mg) was pregnant and died of capture myopathy. While hair samples are assumed to incorporate hormones in circulation during growth, some researchers have raised concerns about the potential for acute stressors to influence cortisol concentrations in hair (Cattet *et al.*, 2014). It is possible that during the acute stress response during capture of this moose, circulating progesterone concentrations increased due to extragonadal sources associated with an acute stress response (Båge *et al.*, 2000) which were incorporated into the hairs around the hooves. Further, Rakic *et al.* (2023) found hair cortisol concentrations in caribou differed between sampling methods with cortisol concentrations being greater in live-captured and sampled animals compared to hair samples collected postmortem from hunted caribou, though the authors could not explain the observed differences.

We observed variation in cortisol concentrations among the guard hair segments from all samples. The cortisol concentrations in guard hair in our study were similar to previous studies and as expected we found variation among hair segments and moose. Previous studies measuring cortisol in moose collected hair samples from the rump or neck (Madslien *et al.*, 2020; Spong *et al.*, 2020), which are shorter and likely grown over a shorter period. Cortisol concentrations in our study were comparable and within the range of values previously reported for adult female moose (Madslien *et al.*, 2020) and concentrations were slightly greater in adult female wild red deer but comparable to our study (Caslini *et al.*, 2016).

### Applications for wildlife management and future considerations

Steroid hormone concentrations in hair samples have largely proven to be a useful tool in studying wild mammal populations (Bryan *et al.*, 2013; Bryan *et al.*, 2014; Koren *et al.*, 2019; Davidson *et al.*, 2021; Pereira *et al.*, 2022), though some questions remain in how steroid hormones are incorporated during growth (Russell *et al.*, 2012). Our findings add to the growing body of literature demonstrating the utility of keratin tissues including claw tissues (Baxter-Gilbert *et al.*, 2014; Crain *et al.*, 2021; Keogh *et al.*, 2022; Roffler *et al.*, 2022). The use of hoof tissue may be particularly useful for cervid populations that require conservation measures to ensure a sustainable resource for hunters and subsistence users as well as allowing researchers to investigate potential factors influencing reproduction in wild cervid populations.

Sampling hooves as outlined in our study, provides biologists and managers with another tool for understanding what variables may contribute to population level changes in reproduction. The application of the growth rates would allow biologists to determine where and how many samples should be collected based on when sampling occurs. This is particularly useful during field efforts on immobilized moose, allowing biologists to limit the time and handling needed to collect samples. Further, the collection of hoof tissue from live or postmortem moose as we describe allows for repeated sampling of hoof tissue that could be used for other studies such as dietary stable isotopes.

## Supplementary Material

Web_Material_coad097

## Data Availability

Data for this project is available upon reasonable request from the Alaska Department of Fish and Game and subject to a data sharing agreement.
