# Supplementary material for: Tracking reproductive events: Hoof growth and steroid hormone concentrations in hair and hoof tissues in moose (Alces alces)
Source: Conserv Physiol. 2023 Dec 13;11(1):coad097. doi: 10.1093/conphys/coad097 (PMC10722880; doi:10.1093/conphys/coad097)
Supplement: Web_Material_coad097 [file web_material_coad097.pdf]

Supplementary Data
Title: Tracking reproductive outcomes: Hoof growth and steroid hormone concentrations in hair and hoof tissues in moose (*Alces alces*)
Journal: Conservation Physiology
Authors: Mandy J. Keogh, Daniel P. Thompson, John. A. Crouse.
Corresponding Author: Mandy J. Keogh, NOAA Fisheries, P.O. Box 21668 Juneau, AK, 99802-1668, USA. email: mandyjkeogh@gmail.com

Supplementary Data S1. Results of added mass for progesterone and cortisol for moose hair and hoof tissues. Briefly, assay accuracy was assessed by spiking standards with an equal volume of pools. Results were plotted as the observed versus expected standard dose and assessed for linearity, slope, and y-intercept.

	Progesterone		Cortisol	
	Paral	accuracy	Paral	accuracy
Guard Hair	-23.77ln(x)+205.85 R^2=0.999	0.989x+210.92 R^2=0.970	-R^2 = 0.9849-- 22.69ln(x)+198.74	0.8638x-10.778 R^2=0.9984
Hoof	-23.88ln(x)+201.5 R^2=0.9932	0.810x+32.788 R^2=0.9947	-18.5ln(x) + 161.68 R^2 = 0.9997	0.9104x-259.63 R^2=0.9010

Supplementary Data S2. (A) Annual body mass curve based on the body mass from adult female MRC moose (n = 410). B) Estimated hoof growth rate in context of body mass change (Daily Hoof growth = Corrected rate of body mass change • 0.1522 + 0.037) and then regressed against cosine(JD) to create a curve (Fig. 2) with the equation Daily hoof growth = -0.116X + 0.1525 where X = cos(radian(JD)).

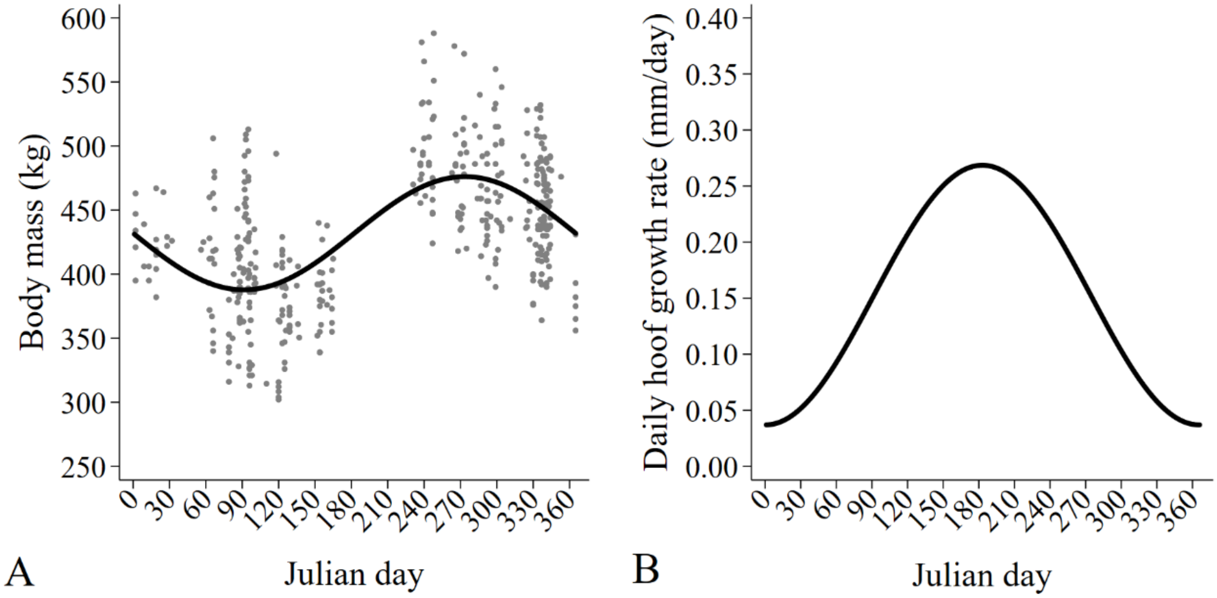

Supplementary Data S3. Growth rate (mm/day) and measured growth from the front hooves from adult female moose (n = 7) at the Kenai Moose Research Center, Kenai Peninsula, Alaska, USA. The estimated hoof growth (mm) is based on body mass from adult female MRC moose (n = 410).

Date Range	Growth Rate (mm/day)	Measured Growth (mm)	Estimated Growth (mm)
8/28 – 12/5	0.1588973	15.89	12.80
12/5 - 4/1	0.0846296	9.82	8.14
4/1- 8/27	0.2049645	30.33	34.83
